# Advances in CRISPR-Cas9 for the Baculovirus Vector System: A Systematic Review

**DOI:** 10.3390/v15010054

**Published:** 2022-12-24

**Authors:** Duygu Sari-Ak, Omar Alomari, Raghad Al Shomali, Jackwee Lim, Deepak B. Thimiri Govinda Raj

**Affiliations:** 1 Department of Medical Biology, Hamidiye International School of Medicine, University of Health Sciences, 34668 Istanbul, Turkey; 2 Hamidiye International School of Medicine, University of Health Sciences, 34668 Istanbul, Turkey; dromari2001@gmail.com (O.A.); raghadalshomali@gmail.com (R.A.S.); 3 Singapore Immunology Network, A*STAR, 8a Biomedical Grove, Singapore 138648, Singapore; lim_jack_wee@immunol.a-star.edu.sg; 4 Synthetic Nanobiotechnology and Biomachines Group, Synthetic Biology and Precision Medicine Centre, Next Generation Health Cluster, Council for Scientific and Industrial Research (CSIR), Pretoria 0001, South Africa; dgovindaraj@csir.co.za

**Keywords:** BEVS, CRISPR-Cas9, MultiBac, Complex Glycosylation

## Abstract

The baculovirus expression vector systems (BEVS) have been widely used for the recombinant production of proteins in insect cells and with high insert capacity. However, baculovirus does not replicate in mammalian cells; thus, the BacMam system, a heterogenous expression system that can infect certain mammalian cells, was developed. Since then, the BacMam system has enabled transgene expression via mammalian-specific promoters in human cells, and later, the MultiBacMam system enabled multi-protein expression in mammalian cells. In this review, we will cover the continual development of the BEVS in combination with CRPISPR-Cas technologies to drive genome-editing in mammalian cells. Additionally, we highlight the use of CRISPR-Cas in glycoengineering to potentially produce a new class of glycoprotein medicines in insect cells. Moreover, we anticipate CRISPR-Cas9 to play a crucial role in the development of protein expression systems, gene therapy, and advancing genome engineering applications in the future.

## 1. Introduction

**Figure 1:**
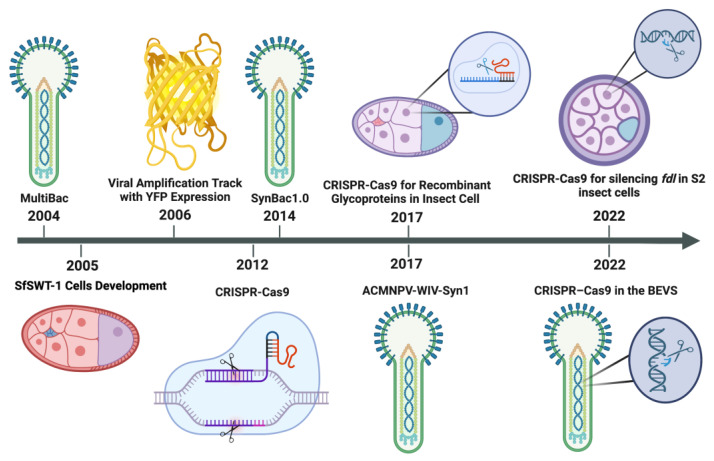
Evolution of Baculovirus expression vector system (BEVS), MultiBac technology and CRISPR/Cas9 system. MultiBac has made it simple to access baculoviral genomes ever since it was first used, in addition to the incorporation of CRISPR components on both natural and synthetic insect cells, and minimized genomes (SynBac). fdl: fused lobes gene; SfSWT-1: commercial transgenic insect cell line called mimic Sf9; YFP: Yellow Fluorescent Protein.

CRISPR has transformed biomedical research over the past ten years and offered whole new methods for investigating every aspect of cell biology. The development of CRISPR and related tools has opened a window into previously unsolvable issues in our understanding of genetics, the noncoding genome and heterogeneity, and provided new insights into therapeutic vulnerabilities for variable diseases. The field of genome editing of various organisms has advanced quickly over the last two decades (Figure 1), and as a result, it has drawn a lot of attention from researchers who have reviewed the most recent developments in-depth and offered their predictions for the field’s future developments. While CRISPR-Cas technologies were developing greatly and very quickly in insects in vivo and cultured cells in vitro, it also helped to develop other genetic editing and protein expression systems such as the baculovirus expression vector system (BEVS). In this review, we discuss baculoviral delivery systems and how current vector systems can be developed through CRISPR gene editing and genome engineering applications.

## 2. Baculovirus Expression Vector System (BEVS)

Baculoviruses are arthropod-specific enveloped viruses with large, double-stranded DNA (dsDNA) genomes that are members of the Baculoviridae family [1]. They infect more than 600 different host species of various orders, particularly insects of the order Lepidoptera [2]. Baculoviruses have a biphasic life cycle during which they produce two distinct virion forms: occlusion-derived virus (ODV) and budded virus (BV). ODV is involved in the primary infection and horizontal transmission of the virus between insect larvae after the ingestion of occlusion bodies (OBs), which have the virus embedded within. BV, on the other hand, is responsible for the secondary infection, involving the spread of infection between tissues and cells within the host larvae [3].

The baculoviruses were first used as an expression system in the mid 1980′s after much improved biological understanding of the prototype baculovirus, Autographa californica multiple nuclear polyhedrosis virus (AcMNPV). In AcMNPV, the OBs are formed from polyhedrin protein, which is highly expressed at the very late stage of infection [4]. Although the polyhedrin protein, which is expressed in the polh locus, is neither required for systemic infection within the larva nor the spread of infection in insect cell culture via BV production [5], by making use of the potent polh promoter, extremely large quantities of target protein could be produced in fall armyworm-derived insect cell cultures. In another pioneering work, the polyhedrin gene (polh) was deleted from the genome of AcMNPV and replaced by DNA-encoding human IFN-beta, which led to the production of high levels of biologically active human β-interferon protein [6]. These works established the BEVS as a powerful and highly regarded eukaryotic protein expression tool, as a result of initial modification to the baculoviral genome and the remarkable production of the recombinant protein. Shortly after this discovery, another study showed that another highly expressed very late gene, p10, similar to polh, could be used for the large-scale production of heterologous recombinant proteins [7].These two constitutional studies demonstrated the effectiveness of the baculovirus/insect cell expression system for recombinant protein production.

The BEVS has become one of the most widely used systems in heterologous protein expression. It has since been used to produce thousands of different recombinant proteins, including the production of virus-like particles (VLPs), including VLPs from bluetongue and rotavirus, to study viral assembly processes, to produce antigens for immunization and diagnostic assays, and even in the manufacturing of commercially available vaccines [8,9,10,11,12]. BEVS has also provided an effective way to overcome some difficulties in the implementation of other expression systems, as it was used to overcome the poor production of recombinant adeno-associated viruses (AAVs), which are now produced in sufficient quantities of AAV vectors for large scale studies [13]. For the generation of baculoviruses, virus-like particles, recombinant proteins, and gene therapy vectors, more than 400 different types of insect cell lines have been developed but the most widely used type is Sf9, which is a clonal isolate of Sf21 cell line derived from pupal ovarian tissue of *Spodoptera frugiperda* [14]. Another commonly used cell line is BT1-TN5B1-4, also known as High Five, which is derived from ovarian tissue of adults from Cabbage Looper Trichoplusia ni [15]. Less often-used cell lines include Bm5, BmN, and Bme21, which were produced from *Bombyx mori* embryos, as well as A7S and DpN1, which were produced from Pseudaletia unipuncta and Danaus plexipus larvae, respectively [16]. The system did not solely rely on the expression of genes in infected insect cells. Rather, it was discovered that it is also possible to transduce mammalian cells with the baculovirus by altering its tropism, which is termed ‘BacMam’ [17,18,19]. Since the insect promotors are inactive in mammalian cells, the transduction allows only the delivery of the expression vector, but not the replication of the virus. Consequently, the BacMam vector system is an intrinsically safe way to transduce mammalian cells, including human cells. To initiate recombinant protein production, BacMam vectors use mammalian-specific regions such as the human cytomegalovirus (CMV) immediate early gene promoter [20]. Through several surface modifications, baculovirus mediated transduction of various human cells including hepatocytes, dendritic cells, monocytes, granulocytes, B lymphocytes, and different human cancer cells was improved [18,21,22,23]. These examples highlight the potential of baculovirus vectors in gene therapy.

Baculovirus has some advantageous factors, making BEVS an outstanding expression system. First is its ability to prioritize its genome expression by arresting host gene transcription [24]. In addition to the virus’ inability to replicate in human cells, the removal of polh or p10 genes, which prevents the formation of OBs and therefore the survival of the virus in nature, increases the biosafety of the system [6]. Furthermore, BEVS does not show the limitations associated with other systems such as AAV, lentivirus, or yeast, enabling the expression of large properly folded heterologous proteins and eukaryotic post-translational modifications without being infectious or hazardous to human health (Table 1) [25]. In contrast to other viruses that have a crystalline shell, baculoviruses have a flexible envelope that can expand to accommodate the increasingly large genome packaged inside the baculovirion without impairing baculovirus function [26]. However, due to its large size, it is challenging to directly introduce genes using traditional molecular cloning techniques into the 130 kb dsDNA-sized baculovirus genome. Instead, the majority of BEVS use an intermediary plasmid vector, into which the foreign gene is cloned, and various techniques to transfer the gene from the plasmid vector into the virus genome [27].

## 3. Development of MultiBac

Proteins rarely function in isolation; protein complexes can consist of several proteins and other biomolecules to perform the key cellular functions. Multiprotein complexes are the cornerstones of biological activity and development, and are essential for cell homeostasis and catalysis of major functions of cells [45,46]. Therefore, the structural and functional analysis of these multiprotein assemblies is crucial to complete our understanding of cell biology in addition to many diseases where the protein complexes have an important role in the pathogenesis. However, the molecular analysis necessitates the purification of the proteins in large enough quantities, which is not possible most of the time due to their low abundance in the native cell environment [47]. Thus, recombinant overproduction offers a solution to this problem by enabling high-level production of homogeneous and active eukaryotic complexes for in-depth molecular analysis. Because of the aforementioned advantageous features of the BEVS, particularly its large DNA cargo capacity, it was specifically engineered for the expression of multiprotein assemblies to be used in structural and functional analysis studies [48,49,50,51]. This multiprotein expression system was called MultiBac, and remains widely used across different research areas [47].

The MultiBac consists of an engineered baculovirus that has been customized for multiprotein complex expression [48]. By removing chiA and v-cath genes, the proteolytic and apoptotic functions are deleted from the baculoviral genome to enhance protein expression by delaying cell lysis and reducing degradation [48,49,50,52]. In addition, the baculovirus genome is utilized as a bacterial artificial chromosome (BAC) in *E. coli* cells. The MultiBac system comprises two types of transfer plasmids, called acceptors and donors [49,51,53]. Donor plasmids contain conditional origin of replication, R6Kγ, only allowing their survival and replication in *E. coli* strains that express their pir gene, whereas acceptor plasmids have standard replicons [50]. The fusion of plasmids is mediated by LoxP/Cre recombinase and desired acceptor–donor fusion products is enabled by antibiotic resistance markers [41], to generate bacmids using the EMBacY MultiBac system (Geneva Biotech, Geneva, Switzerland). EmBacY competent *E. coli* which contains both the bacmid and helper plasmid are selected based on blue/white screening (white colonies represent successful disruption of the LacZα gene by the gene-of-interest). The recombinant bacmid is then extracted, purified and transfected into insect cells, after which standard protocols are used for virus amplification and protein expression [49,52].

To facilitate the use of these systems, several improvements and modifications have been applied. One improvement includes ExpiFectamine Sf (Thermo Fisher Scientific, Waltham, MA, USA), a next-generation cationic lipid based reagent for more robust transfection, and modifications include using RNAi technology or CRISPR Cas9 for engineering genome composition, which led to improved expression process and helped in overcoming some of the limitations of the BEVS [14].

With advancement of synthetic biology technologies and development of minimal genome, different techniques such as homologous recombination were applied to re-engineer baculovirus genome. Both top-down and bottom-up approaches have been implemented for reengineering of baculovirus genome. Shang Yu et al. developed a fully functional synthetic baculovirus designated AcMNPV-WIV-Syn1 using the combination of PCR, transformation and homologous recombination in yeast [54]. Thus, Shang Yu et al. demonstrated that proof-of-concept synthetic baculovirus genome can be generated. Further, Shang Yu et al. developed a novel bacmid AcBac-Syn by combining the vector containing LacZ: attTn7 along with eGFP cassette and recombined with a linearized AcMNPV-WIV-Syn1 genome [55]. Although novel bacmid AcBac-Syn was functional, there is no clearly demonstrated evidence of superior properties of AcBac-Syn compared to existing BEVS (Multibac, Bac-to-bac and other expression system). In order to improve the cargo capacity and address the scale-up bottleneck of BEVS, SynBac1.0 was developed, where 10 kb of non-essential genes (~8% of the ACMNPV genome) were removed through homologous recombination [56]. SynBac1.0 demonstrated high protein expression and higher stability compared to MutliBac [26]. Further rewiring of SynBac1.0 has been implemented using homologous recombination in order to remove other non-essential genes and demonstrate higher protein expression [26]. Development of reduced synthetic genomes such as SynBac shows tremendous potential where Cas9 machinery can be potentially integrated into the baculovirus backbone.

## 4. CRISPR/Cas9 Technology

In recent years, the Clusters of Regularly Interspaced Palindromic Repeats (CRISPR)/Cas system has become a potent tool for genome editing. The CRISPR system is naturally present in a large variety of bacterial species to provide a rich source of functional diversity for genome editing in eukaryotic cells, [57,58]. Ishino et al. first discovered CRISPR as repeating palindromic repeat sequences separated by 32 base-pair sequences in 1987 [59]. Further research into CRISPRs revealed it to be part of the bacterial defense immune system against invading viruses, and the programmability of Cas9 through CRISPR RNA (crRNA) allowed for its use as a genetic editing tool [60,61,62]. The CRISPR-associated nuclease (Cas) system is a widely distributed acquired defense system in many bacteria and archaea that shields organisms against invading viruses and plasmids [62,63,64]. There has been a significant expansion of variant enzymes that increase the functionality of CRISPR-based platforms since the original application of CRISPR systems in eukaryotic cells. The wide variety of Cas9 orthologues, including Staphylococcus aureus Cas9 (SaCas9) and other Cas enzymes (such as Cas12), found in a variety of bacterial species, is considered one of the most important sources of these variations [58,65]. Each has a unique collection of sequence recognition features and standards, which gives it more adaptability when used as a tool for research or treatment [66]. Presently, three processes are known to make up the CRISPR-Cas immune response: adaptation, expression and processing of pre-CRISPR RNA (pre-crRNA), and interference to prevent prokaryotes from becoming infected [67,68]. Moreover, the CRISPR/Cas system has two main classes; these two systems are further separated into six kinds and 33 subtypes, each of which contains a distinctive Cas gene [69]. Because of its great efficacy, and simplicity of usage, the type II CRISPR/Cas9 system is mostly used in gene editing [70]. A wide range of species and cell types, including human cells, bacteria, mice, fruit flies, yeast, zebrafish, roundworms, rats, and pigs have been successfully genetically edited with the help of the Streptococcus pyogenes Cas9 (SpCas9), which recognizes the 5′-NGG-3′ protospacer adjacent motif (PAM) sequence, where N is any nucleotide [71]. The range of genetically tractable model species is also being considerably expanded by SpCas9 day by day.

Cas9 is a multifunctional protein with two putative nuclease domains, HNH and RuvC [72]. HNH and RuvC have the ability to cleave both the complementary strand and non-complementary strand of DNA, respectively. Additionally, a chimeric sgRNA is needed to activate the CRISP/Cas9 DNA repair machinery, which is composed of a target specific 17-20-nucleotide CRISPR RNA (crRNA) and a 85-nucleotide transactivating CRISPR RNA (tracrRNA), which stabilizes the ribonucleoprotein complex system (RNPC) [73,74,75]. DNA repair machinery is initiated in the order of PAM recognition, sgRNA binding and RNPC formation, which direct the two catalytic nuclease domains to induce double-stranded DNA break (DSB) ~3 to 4-nucleotide upstream of the PAM site. Therefore, by designing specific crRNA that recognizes the viral DNA sequence, Cas9 is able to target DNA in the genome, initiate DSBs and interfere with the production of the target genes [72,76].

CRISPR and Cas proteins have revolutionized genomic engineering through novel applications, such as the investigation of developmental pathways, gene expression regulation, and animal behavior. In addition, CRISPR/Cas9 has also been used to recruit functional domains that alter gene expression or mark specific genomic regions in organisms or living cells. However, genome engineering is limited in realizing large-scale genome editing with numerous genes or large DNA fragments due to the relatively complicated process for creating DNA-editing templates. In 2016, due to the rapid and efficient inactivation of bacterial gene(s) in a homologous recombination-independent manner, a CRISPR-Cas9-assisted non-homologous end-joining (CA-NHEJ) method has been described [77]. Large chromosomal DNA fragments could now be deleted using the NHEJ method in a single step without the need for a homologous DNA template thanks to CRISPR-Cas9.

Different DNA damage repair processes, including classical non homologous end joining (cNHEJ), homology-directed repair (HDR) and microhomology-mediated end joining (MMEJ), are used by cells to fix DSBs [78,79]. Finally, asymmetric repair may result from the use of different DNA repair mechanisms to fix each end of a DSBs [80]. Nowadays, these mechanisms are applied in clinical practice in order to relieve or even cure diseases. In conclusion, DSBs are created and repaired at specific locations in CRISPR/Cas9-mediated targeted gene segments. Thus, CRISPR/Cas9 gene editing tool has demonstrated enormous potential in genetic knock-out engineering and has acquired a promising accomplishment by editing the targeted mutation and enhancing the quality of other gene therapy methods.

## 5. CRISPR/dCas9 Technology

In recent years, the inactive variant of spCas9 dead Cas9 (dCas9) has expanded the utility of CRISPR/Cas9. The dCas9 is produced by nullifying both the RuvC and HNH endonuclease domains in Cas9 via D10A and H840A mutations, respectively [81]. Initially, dCas9 was employed as a reversible gene targeting technology through (i) targeted expression of any gene, or many genes by employing multiple guide RNA expressions, and (ii) constitutive or conditional doxycycline-inducible expression of sgRNA [82,83].

More recently, as a chimeric dCas9-X molecule (where X is any functionally active protein), it can recruit modifying enzymes and reporter proteins to DNA target locations for wider usage in genomic visualization, gene regulation and epigenetic modification [84,85]. By combining the precise DNA recognition of dead Cas9 with the Krüppel-associated box (KRAB) repressor, which prevents the transcription of target genes, CRISPR has emerged as an effective tool not just for gene editing applications but also the use of artificial transcriptional regulators [57,81,83]. In the case of manipulating gene expression, the CRISPR/dCas9 system and a transcriptional effector fused to dCas9 or sgRNAs are used, whereby the transcriptional effector can stimulate transcription (CRISPR activation; CRISPRa, CRISPR interference; CRISPRi) [86]. To activate or inhibit a gene of interest (GOI), sgRNAs should be targeted to the promoter region of the GOI. For example, in CRISPRi, the chimeric dCas9, sgRNA, transcriptional effector complex and RNA polymerase are recruited to gene sequences to cause transcriptional interference like RNAi. While both CRISPRi and RNAi aim to inhibit or silence gene expression, they do so through various mechanisms and guiding ideologies [87]. In essence, RNAi uses a post-transcriptional mechanism by cleaving transcribed mRNAs, whereas CRISPRi suppresses gene expression at a DNA level by inhibiting transcription.

Today, when compared to other genomic techniques, the CRISPR/dCas9 technology is a more straightforward, convenient, effective, and fairly priced technique for selectively activating or repressing gene expression [88]. However, the simultaneous delivery of many components into living cells using CRISPR-based precision gene editing can exceed the cargo capacity of conventional viral vector systems. To enable the multiplexability of genome engineering, the unique heterologous DNA cargo capacity of baculovirus was thus used to solve this issue in human cells, and provide a versatile delivery platform for single base to multi-gene level genome interventions [89]. Aulicino et al. has successfully completed whole-exon replacement in the intronic β-actin (ACTB) locus by encoding Cas9, sgRNA, and donor DNAs on a single, quickly assembled baculoviral vector [89]. These results demonstrate the efficacy of baculovirus-vectored approach to overcome cargo limitation and tackle the CRISPR delivery challenge.

## 6. Insect Cell Line Modification Using CRISPR/Cas9

Besides mammalian cells, CRISPR-Cas9 can also provide precise genetic modification of the BEVS to enhance its ability of producing recombinant proteins for structural analysis and use in biomedical applications [90,91]. Some baculoviruses can stimulate the secretion of anti-apoptotic proteins, such as p35, through insect cells, including Sf9 cell lines, after invading it, which inhibits the death of the cells and helps in producing higher levels of recombinant proteins [92,93]. However, it was found that insect cells possess innate defense mechanisms against these baculoviruses [94]. Therefore, a vector called multiple editing anti-BmNPV therapeutic complex CRISPR-Cas9 system was developed by Dong et al. that can disrupt the replication of BmNPV [95]. This enables multiplex genome engineering by CRISPR/Cas9 in baculoviruses and probably the development of antiviral therapy.

In 2021, the usage of the CRISPR/Cas9 system for baculovirus genome editing was investigated. To demonstrate the effectiveness of targeted gene disruption (CRISPRd) and repression (CRISPRi) in BEVS, Bruder et al. found that CRISPRd is more efficient than CRISPRi in complete disruption of target genes in transgenic Sf9 cell line bearing Cas9, making it suitable for characterizing non-essential endogenous baculovirus genes to reduce baculovirus contamination in culture supernatant [96]. In contrast, CRISPRi still permits expression of the targeted gene at levels lower than the wildtype, making it more useful in providing higher production yield of recombinant protein therapeutics by prolonging the infection cycle of the BEVS [96].

Besides higher production yield, potential industrial applications of high-performance optimized insect cell lines include using it as a pest control agent [97]. Pazmiño-Ibarra et al. used CRISPR/Cas9 for the first time for genome editing in baculovirus to improve the protein expression capacity of BEVS and the use of baculovirus as a biopesticide. By targeting the egt locus, they were able to produce non genetically modified (non-GM) viruses that can be used in pest control. The knockout of the egt gene was shown to enhance the insecticidal properties of baculovirus in previous studies [98,99,100,101].

With the advancement of CRISPR/Cas9 technology along with BEVS capability to carry large cargo, the BacMam system has been applied for CRISPR/cas9 delivery for highly efficient gene editing in human cells [58]. With significant development towards base and prime editing technologies for addressing unmet medical need such as blood disorders [102,103], BacMam has strong role to play as a CRISPR/cas9 delivery system in human cells. BacMam has been used as a CRISPR/cas9 delivery system for several applications, ranging from CRISPR-mediated tagging of proteins [104], simultaneous delivery of CRISPRs/cas9 gene editing machinery and drug release as aa nanodevices for biomedical treatment [105], precise docking of large multifunctional DNA circuits [106], and precise gene delivery to mammalian cells [107].

## 7. Glycosylation

Proteins typically undergo post-translational modifications (PTMs) after translation, which are essential for proteins to function normally in the body. These PTMs include acetylation, ubiquitination, glycosylation, and phosphorylation. Glycosylation is one of the most important common and intricate PTM of numerous cell surface and secreted eukaryotic proteins. In the endoplasmic reticulum (ER), oligosaccharides are enzymatically added to developing polypeptide chains by glycosylation. Additional modifications to the attached oligosaccharide structure are made by a variety of glycosidases and glycosyltransferases found in the ER and Golgi complex [108]. The modification reactions that take place in ER are substantially conserved higher and lower eukaryotes. However, modification reactions that occur within the Golgi complex vary depending on the species and kind of cell [109]. A glycan’s biological activity can be directly modulated by specific structural changes; therefore, numerous biological functions depend heavily on glycosylation. Glycoproteins make up around 50% of natural human proteins, including immunoglobulin [110]. In addition to being crucial for glycoprotein folding, glycans are implicated in disease conditions, maintaining cellular homeostasis, and regulating the immune system [110]. Glycans affect biology in three different ways. First, by way of their physicochemical characteristics, which range from constituting integral parts of the extracellular matrix to supporting protein folds [111,112]. Second, the characteristics of the protein or lipid to which they are bound can be modified by glycans [113]. Third, they are recognized by glycan-binding proteins (GBPs) [113]. Glycan recognition by GBPs is crucial for cellular communication and cell transport [114].

## 8. Glycosylation in BICS

As already indicated, BEVS and insect cell system has been utilized to create hundreds of recombinant proteins, ranging from membrane-bound proteins to cytosolic enzymes. BEVS system may be utilized to create recombinant proteins with different O- and N-glycan structures, excluding the step of sialylation [115]. However, many glycoproteins, like EPO and certain antibodies, as well as their biological activity and their serum half-life, depend on sialylation [116]. Thus, the broad application of glycoprotein biological products of BEVS is restricted because of these limitations. In fact, the most abundant sialic acid nucleotide, cytidine monophosphate (CMP)-sialic acid (CMP-Neu5Ac), which is a necessary substrate for sialyltransferases, is marginally produced by insect cells [108]. More importantly, the development of insect cell lines that express mammalian genes for proteins with N-glycan processing activity has received a significant amount of interest in the last two decades. Further, BacMam systems has been applied for high level expression of recombinant membrane glycoprotein expression in mammalian cell systems. Recently, highly glycosylated porcine Interferon Alpha expressing BacMam has been applied as antiviral activity against foot-and-month disease virus in veterinary space [117].

One of the major limitations associated with the expression of proteins that affect the normal functioning of the expressed proteins is the inability of insect cells to perform N-glycosylation in the same way as mammalian cells. N-glycosylation is one of the most important post-translational modifications (PTMs) that involves the addition of terminal galactose and sialic acid. Due to the lack of glycogen or glycosidase activity, this PTM cannot be carried out in insect cells [118,119]. Now, CRISPR-Cas9 tools for site-specific genome editing are used to alter protein glycosylation in the baculovirus–insect cell system (BICS).

## 9. Transgenic Insect Cells for BICS

A commercial transgenic insect cell line called mimic Sf9 (also known as SfSWT-1) was developed from Sf9 cells and is designed to produce highly processed, mammalian-like recombinant proteins with terminally sialylated N-glycans [120]. Five mammalian glycosyltransferases that mimic Sf9 are integrated, resulting in recombinant proteins that are more glycosylated than those produced by Sf9 or High Five cells [120].

Five mammalian glycosyltransferases (SfSWT-1; human β1, rat α2,6- sialyltransferase (ST6Gal I), mouse α2,3-sialyltransferase IV (αST3Gal IV), 2-N acetylglucosaminyltransferase I and II, (GlcNAc-T I, GlcNAc-T II) and bovine β1,4-galactosyltransferase (β4Gal-T I) have been integrated into Sf9 cell lines that express a variety of mammalian genes for N-glycan processing [121]. Bi-antennary, terminally sialylated N-glycans could be produced by SfSWT-1 cells [122]. This cell line was enhanced to encode two extra mammalian (mouse) genes for the CMP-sialic acid synthetase (CMAS) and sialic acid synthase (SAS), resulting in the development of of SfSWT-3 [123], which generates CMP-sialic acid and produces recombinant sialylated glycoprotein when incubated in a serum-free medium containing N-acetylmannosamine. Other SfSWT-4 and SfSWT-5 cell lines, when cultured with sialic acid precursor N acetylmannosamine, with the bare minimum set of genes, were also able to produce terminally-sialylated N -glycans from constitutive or inducible promoters [124,125]. The human CMP-sialic acid transporter (hCSAT) was added to SfSWT-4 during subsequent engineering, resulting in SfSWT-6, which supports higher levels of recombinant glycoprotein sialylation when incubated in low concentrations of N-acetylmannosamine and leads to larger amounts of detected sialylation [125].

## 10. The Future of CRISPR/Cas9 to Complex Glycosylation in BICS

Efforts have been made to rewire the N-glycosylation pathways of insect cell lines to be mammalian-like by utilizing non-homologous recombination. Unfortunately, the ability of non-homologous recombination is constrained, and additional glycoengineering is needed for the insect cell host system to manufacture mammalian type glycoproteins. Nonetheless, the CRISPR-Cas9 systems appear to be a possible solution. Previously, the protein glycosylation processes in *Drosophila melanogaster* were rewired using CRISPR/Cas9 gene editing. Both the S2R+ cell line, derived from *Drosophila melanogaster* (Dm), and the insect cell line, derived from *Bombyx mori* (Bm), have undergone gene editing using the CRISPR-Cas9 system. Despite many examples of CRISPR/Cas9 genome engineering, there are still not enough studies and published data in the literature about utilizing CRISPR/Cas9 for modifying protein glycosylation processes or gene editing in insect cells. Nonetheless, there is potential for using CRISPR/Cas9 gene editing in insect cells given the current demand for high yield synthesis of protein therapies derived from insect cells that can address unmet medical needs. With the help of CRISPR/Cas9 gene editing, it may become possible to create insect cell lines to produce therapeutic recombinant human proteins that are effectively glycosylated and mimic the structure of human glycans.

Of note, a technique for CRISPR/Cas9 gene editing in Sf9 insect cells was first performed by Donald L. Jarvis and colleagues [126]. The team used the CRISPR-Cas9 vector as a platform to produce recombinant glycoproteins [127]. They also evaluated the potential for site-specific gene editing in baculovirus insect cell system (BICS) host insect cell lines by using several types of insect cell U6 promoters upstream of gene cassette to create CRISPR-Cas9 expression vector in Sf9 and High Five cells. The generic CRISPR-Cas9 vectors with DmU6 or BmU6-2 promoters did not allow CRISPR-Cas9 gene editing in Sf9 cells. However, the ability to manufacture indels was confirmed by sequencing using the CRISPR-Cas9 vector that has a SfU6-3 promoter (rather than DmU6 or BmU6-2) [127].

Both mammalian and insect cells can produce similar glycan-intermediates. However, mammalian cells extend this intermediate to complex N glycans with various glycosyltransferases, while insect cells cannot elongate this product due to a membrane-bound enzyme, N-acetylglucosaminidase, which catalyzes the removal of terminal N-acetylglucosamine residue in trimmed N-glycan processing intermediates [128]. Similarly, two Lepidopteran insect cell lines have been shown to transfer and trim nascent polypeptides to yield the same processing intermediates as mammalian cells [14]. However, the N-glycosyl intermediate is eliminated as a substrate for N-acetylglucosamine transferase II by N-acetylglucosaminidase, terminating as simple paucimannosidic N-glycans in insect cells [14]. As a solution, the fdl gene, which encodes for N-acetylglucosaminidase, was recently deleted in Sf9 cells, thus promoting further elongation toward homogenous, terminally sialylated N-glycans [126]. Through RNA interference, silencing the fdl gene has also been attempted, thereby inhibiting the activity of this enzyme (RNAi) [128,129]. Recently, it has been shown that the CRISPR-Cas9 system can silence the fdl gene in S2 cells and BmN4-SID1 insect cells. Indeed, CRISPR-Cas9 effectively produced insect-type paucimannose products and glycoproteins with a complex-type N-glycan structure, respectively [130,131]. For example, S2 cells generated mostly elongated complex N-glycans of the mammalian type, ranging from Man5 to Man9 [130]. According to Mabashi et al., site-specific genome editing of the fdl gene was accomplished using CRISPR-Cas9 in Sf9 or BmN cells by producing a functional Cas9 under the control of the IE1 promoter and functional sgRNAs under the control of the DmU6:96Ab and BmU6-2 promoters [126]. These results indicate the possibility of using a CRISPR-Cas9 system to silence genes such as fdl gene to rewire glycosylation processes, which could drive the development of a new class of glycoprotein medicines with specialized functions.

## 11. Conclusions

BEVS is now one of the most widely used vector expression systems for producing recombinant proteins for structural analysis and biomedical applications. Several improvements, including MultiBac, BacMam and MultiBacMam systems, have been developed to diversify the range of BEVS-related applications. Although the BEVS has many advantages compared with other expression systems, it does have some limitations that affect the production of certain glycosylated proteins. One of the main limitations is the insect cells’ inability to perform glycosylation the same way as mammalian cells. Nonetheless, advanced bioengineering tools such as iRNA and CRISPR/Cas9 have proven to be very promising solutions to diversify protein type expression by BEVS. We are now able to use CRISPR/Cas9-modified insect cell lines to perform mammalian cell-like glycosylation. Additionally, by applying CRISPR/Cas9 or RNAi technology to enable the baculovirus vector to acquire anti-apoptotic capabilities, it may be possible to increase expression level of heterologous proteins in longer surviving insect cells. Taken together, applying RNAi and CRISPR/Cas9 technologies to develop transgenic cell lines and recombinant baculoviruses can broaden and enhance the utility of BEVS in manufacturing proteins.

## Figures and Tables

**Table 1:** Advantages and Disadvantages of Different Expression Systems. Adenovirus [28,29,30], AAV [31,32,33], Retrovirus [29,34,35], Lentivirus [29,32,36,37], HSV [29,38], Poxvirus [29,30,39], Baculovirus [12,40,41], Yeast [40,42], *Escherichia coli* (*E. coli*) [43], *Drosophila melanogaster* [44], *Lactobacillus zeae* [44].

Vector System	Advantages	Disadvantages
**Adenovirus**	Large quantities of high-titer viral stocks can be produced easily (10^10^ pfu/mL)	No integration into host cell genome can also be an advantage since errors related to random insertion are avoided
	Ability to infect both dividing and non-dividing cells	High levels of pre-existing immunity in humans
	Non-oncogenic	Highly immunogenic
	Good insert capacity (carry up to 8 kbp)	Transient gene expression
**Adeno-Associated Virus**	Highly safe since they have never been shown to cause any human disease	Small packaging size (~5.0 kb, including inverted terminal repeats; ITRs)
	Some serotypes have the capacity to bypass the blood–brain barrier (BBB), allowing for the transduction of the central nervous system (CNS) via systemic administration to be used as vector for gene therapy in neurodegenerative diseases.	Slow onset of gene expression, due to the requirement of conversion of the single-stranded AAV DNA into double- stranded DNA
	Broad host and cell type tropism range	They persist as non-replicating episomes and are therefore gradually lost in mitotic cells
	Have the ability to transduce both dividing and non-dividing cells	
	High levels of gene expression for long-term (over years)	
	Heat stability and resistance to solvents and changes in pH and temperature	
	Low immunogenicity and cytotoxicity	
Retrovirus	Non-immunogenic	They have relatively small carrying capacity
	Wide range of target species and cells	Unable to infect non-dividing cells
	Become a permanent part of the host cell genome allowing for stable expression	Random integration into host chromosome, resulting in possible insertional mutagenesis or oncogene activation
Lentivirus	The vector genome integrates into the host cell genome stably leading to long term expression of the transgene	Non-specific integration in the host genome may lead to insertional mutations
	Relatively large carrying capacity (~12–15 kbp)	Uncertainty of biosafety
	Capable of infecting a wide variety of dividing and non-dividing cells	
	The normal function of infected cells is not affected both in vitro and in vivo	
	Enhanced proneness to transduce terminally differentiated tissues from neuronal origin	
**Herpes-Simplex Virus**	Have natural tropism for neuronal cells	Possible cytotoxicity (low safety)
	Vector particles are easily obtained in high titers from tissue culture (10^12^ pfu/mL)	High level of pre-existing immunity in humans
	Can accommodate large amounts of foreign DNA (~50 kbp)	Transient expression of the transgene
	Establish a latent infection during which the viral genome persists indefinitely without any discernible adverse effects on the host cell	The vector genome does not integrate into the host cell genome (can also be an advantage since errors related to random insertion are avoided)
Baculovirus	Baculovirus arrests most host gene transcription, thus prioritizing viral gene expression.	Transient expression of heterologous gene
	Inherent biosafety since the virus does not infect human cells	The bioactivity and immunogenicity of insect expression products are somewhat different from those of the natural product because insect and mammalian cells differ in their glycosylation patterns
	They have flexible capsid and envelope which simply increase in size proportional to the genomic DNA they harbor	
	Because it infects insect cells, BEVS affords eukaryotic post-translational modifications and folding of heterologous proteins.	
	High levels of recombinant protein production	
	BEVS manufacturing is cost-efficient	
Yeast	Cost-effective	Hypermannosylation
	Rapid growth in culture leading to high yield production of proteins	Cannot perform N- and O-linked glycosylation the same way as mammalian cells
	Share many features with higher eukaryotes allowing for protein processing similar to mammalian cells	
	Some intracellularly synthesized proteins to be secreted into the extracellular environment due to the enriched endomembrane system	
	Can produce correctly folded recombinant proteins that have undergone all the post-translational modifications that are essential for their functions	
	Easy to culture and manipulate	
	Safe systems	
***Escherichia coli***	Rapid expression	Proteins with disulfide bonds difficult to express.
	High yields	Produce unglycosylated proteins.
	Ease of culture and genome modifications	Acetate formation resulting in cell toxicity.
	Inexpensive	Proteins produced with endotoxins.
	Mass production is fast and cost effective	Proteins produced as inclusion bodies, are inactive; require refolding.
**Drosophila** **melanogaster**	Quick turn round time	Regulatory records are less than other expression systems
	The protein expressed in its native form	
	No endotoxin release from host cell organism	Possible mammalian virus infection
	Less expensive than mammalian culture	
	Integration of DNA of interest is very stable	Expensive compared to *E. coli* and yeast expression
	Safer than working with mammalian cell lines	
	Usually expresses and secretes even complex post-transitional modified proteins	Proteases present in the cells degrade the protein of interest
	Minor secretion of host cell proteins	
	The vectors are not pathogenic to human	Characteristic N-linked glycan structures of proteins are different when compared to typical mammalian proteins
	Extra-cellular expression to low viscosity medium of correctly folded protein	
*Lactobacillus zeae*	Adapted to temperature sensitive products	No post-translational modifications

## Data Availability

Not applicable.

## References

[1] Possee R.D., Griffiths C.M., Hitchman R.B., Chambers A., Murguia-Meca F., Danquah J., Jeshtadi A., King L.A., Asgari S., Johnson K.N. (2010). Baculoviruses: Biology, replication and exploitation. Insect Virology.

[2] Jehle J.A., Lange M., Wang H., Hu Z., Wang Y., Hauschild R. (2006). Molecular Identification and Phylogenetic Analysis of Baculoviruses from Lepidoptera. Virology.

[3] Rohrmann G.F. (2013). Baculovirus Molecular Biology.

[4] Slack J., Arif B.M. (2006). The Baculoviruses Occlusion-Derived Virus: Virion Structure and Function. Adv. Virus Res..

[5] Summers M.D. (2006). Milestones Leading to the Genetic Engineering of Baculoviruses as Expression Vector Systems and Viral Pesticides. Adv. Virus Res..

[6] Smith G.E., Summers M.D., Fraser M.J. (1983). Production of Human Beta Interferon in Insect Cells Infected with a Baculovirus Expression Vector. Mol. Cell. Biol..

[7] Pennock G.D., Shoemaker C., Miller L.K. (1984). Strong and Regulated Expression of *Escherichia coli* Beta-Galactosidase in Insect Cells with a Baculovirus Vector. Mol. Cell. Biol..

[8] Kost T.A., Condreay J.P., Jarvis D.L. (2005). Baculovirus as Versatile Vectors for Protein Expression in Insect and Mammalian Cells. Nat. Biotechnol..

[9] Noad R., Roy P. (2009). Bluetongue Vaccines. Vaccine.

[10] Pérez de Diego A.C., Athmaram T.N., Stewart M., Rodríguez-Sánchez B., Sánchez-Vizcaíno J.M., Noad R., Roy P. (2011). Characterization of Protection Afforded by a Bivalent Virus-Like Particle Vaccine against Bluetongue Virus Serotypes 1 and 4 in Sheep. PLoS ONE.

[11] Vicente T., Roldão A., Peixoto C., Carrondo M.J.T., Alves P.M. (2011). Large-Scale Production and Purification of VLP-Based Vaccines. J. Invertebr. Pathol..

[12] Felberbaum R.S. (2015). The Baculovirus Expression Vector System: A Commercial Manufacturing Platform for Viral Vaccines and Gene Therapy Vectors. Biotechnol. J..

[13] Sandro Q., Relizani K., Benchaouir R. (2019). AAV Production Using Baculovirus Expression Vector System. Viral Vectors Gene Ther..

[14] Hong M., Li T., Xue W., Zhang S., Cui L., Wang H., Zhang Y., Zhou L., Gu Y., Xia N. (2022). Genetic Engineering of Baculovirus-Insect Cell System to Improve Protein Production. Front. Bioeng. Biotechnol..

[15] Krammer F., Schinko T., Palmberger D., Tauer C., Messner P., Grabherr R. (2010). Trichoplusia Ni Cells (High FiveTM) Are Highly Efficient for the Production of Influenza A Virus-like Particles: A Comparison of Two Insect Cell Lines as Production Platforms for Influenza Vaccines. Mol. Biotechnol..

[16] Palomares L.A., Joosten C.E., Hughes P.R., Granados R.R., Shuler M.L. (2003). Novel Insect Cell Line Capable of Complex N-Glycosylation and Sialylation of Recombinant Proteins. Biotechnol. Prog..

[17] Carbonell L.F., Klowden M.J., Miller L.K. (1985). Baculovirus-Mediated Expression of Bacterial Genes in Dipteran and Mammalian Cells. J. Virol..

[18] Hofmann C., Sandig V., Jennings G., Rudolpht M., Schlag P., Strauss M. (1995). Efficient Gene Transfer into Human Hepatocytes by Baculovirus Vectors. Proc. Natl. Acad. Sci. USA.

[19] Boyce F.M., Bucher N.L. (1996). Baculovirus-Mediated Gene Transfer into Mammalian Cells. Proc. Natl. Acad. Sci. USA.

[20] Possee R.D., Chambers A.C., Graves L.P., Aksular M., King L.A. (2020). Recent Developments in the Use of Baculovirus Expression Vectors. Curr. Issues Mol. Biol..

[21] Mäkelä A.R., Matilainen H., White D.J., Ruoslahti E., Oker-Blom C. (2006). Enhanced Baculovirus-Mediated Transduction of Human Cancer Cells by Tumor-Homing Peptides. J. Virol..

[22] Ge J., Huang Y., Hu X., Zhong J. (2007). A Surface-Modified Baculovirus Vector with Improved Gene Delivery to B-Lymphocytic Cells. J. Biotechnol..

[23] Martyn J.C., Cardin A.J., Wines B.D., Cendron A., Li S., Mackenzie J., Powell M., Gowans E.J. (2009). Surface Display of IgG Fc on Baculovirus Vectors Enhances Binding to Antigen-Presenting Cells and Cell Lines Expressing Fc Receptors. Arch. Virol..

[24] Nobiron I., O’Reilly D.R., Olszewski J.A. (2003). Autographa Californica Nucleopolyhedrovirus Infection of *Spodoptera frugiperda* Cells: A Global Analysis of Host Gene Regulation during Infection, Using a Differential Display Approach. J. Gen. Virol..

[25] Tsai C.-H., Wei S.-C., Lo H.-R., Chao Y.-C. (2020). Baculovirus as Versatile Vectors for Protein Display and Biotechnological Applications. Curr. Issues Mol. Biol..

[26] Pelosse M., Crocker H., Gorda B., Lemaire P., Rauch J., Berger I. (2017). MultiBac: From Protein Complex Structures to Synthetic Viral Nanosystems. BMC Biol..

[27] Chambers A.C., Aksular M., Graves L.P., Irons S.L., Possee R.D., King L.A. (2018). Overview of the Baculovirus Expression System. Curr. Protoc. Protein Sci..

[28] Von Seggern D.J., Nemerow G.R. (1999). Adenoviral vectors for protein expression. Gene Expression Systems.

[29] Warnock J.N., Daigre C., Al-Rubeai M. (2011). Introduction to Viral Vectors. Methods Mol. Biol..

[30] Vannucci L., Lai M., Chiuppesi F., Ceccherini-Nelli L., Pistello M. (2013). Viral Vectors: A Look Back and Ahead on Gene Transfer Technology. New Microbiol..

[31] dos Coura R.S., Nardi N.B. (2007). The State of the Art of Adeno-Associated Virus-Based Vectors in Gene Therapy. Virol. J..

[32] Zheng C.-X., Wang S.-M., Bai Y.-H., Luo T.-T., Wang J.-Q., Dai C.-Q., Guo B.-L., Luo S.-C., Wang D.-H., Yang Y.-L. (2018). Lentiviral Vectors and Adeno-Associated Virus Vectors: Useful Tools for Gene Transfer in Pain Research. Anat. Rec..

[33] Wang D., Tai P.W.L., Gao G. (2019). Adeno-Associated Virus Vector as a Platform for Gene Therapy Delivery. Nat. Rev. Drug Discov..

[34] Anson D.S. (2004). The Use of Retroviral Vectors for Gene Therapy-What Are the Risks? A Review of Retroviral Pathogenesis and Its Relevance to Retroviral Vector-Mediated Gene Delivery. Genet. Vaccines Ther..

[35] Kim S.H., Robbins P.D. (2007). Retroviral vectors for gene therapy. Lysosomal Storage Disorders.

[36] Torashima T., Okoyama S., Nishizaki T., Hirai H. (2006). In Vivo Transduction of Murine Cerebellar Purkinje Cells by HIV-Derived Lentiviral Vectors. Brain Res..

[37] Meunier A., Pohl M. (2009). Lentiviral Vectors for Gene Transfer into the Spinal Cord Glial Cells. Gene Ther..

[38] Lachmann R. (2004). Herpes Simplex Virus-Based Vectors. Int. J. Exp. Pathol..

[39] Guo Z.S., Bartlett D.L. (2004). Vaccinia as a Vector for Gene Delivery. Expert Opin. Biol. Ther..

[40] Yin J., Li G., Ren X., Herrler G. (2007). Select What You Need: A Comparative Evaluation of the Advantages and Limitations of Frequently Used Expression Systems for Foreign Genes. J. Biotechnol..

[41] Gorda B., Toelzer C., Aulicino F., Berger I. (2021). The MultiBac BEVS: Basics, Applications, Performance and Recent Developments. Methods Enzymol..

[42] Ma Y., Lee C.-J., Park J.-S. (2020). Strategies for Optimizing the Production of Proteins and Peptides with Multiple Disulfide Bonds. Antibiotics.

[43] Biswas K., Pandey P., Kumar R., Maurya M. (2016). Microbial recombinant protein: An epic from fundamental to future panorama of life science. Research Trends in Molecular Biology.

[44] Jarvis D.L. (2009). Baculovirus-Insect Cell Expression Systems. Methods Enzymol..

[45] Robinson C.V., Sali A., Baumeister W. (2007). The Molecular Sociology of the Cell. Nature.

[46] Bieniossek C., Berger I. (2009). Towards Eukaryotic Structural Complexomics. J. Struct. Funct. Genom..

[47] Sari D., Gupta K., Thimiri Govinda Raj D.B., Aubert A., Drncová P., Garzoni F., Fitzgerald D., Berger I. (2016). The MultiBac baculovirus/insect cell expression vector system for producing complex protein biologics. Advanced Technologies for Protein Complex Production and Characterization.

[48] Berger I., Fitzgerald D.J., Richmond T.J. (2004). Baculovirus Expression System for Heterologous Multiprotein Complexes. Nat. Biotechnol..

[49] Fitzgerald D.J., Berger P., Schaffitzel C., Yamada K., Richmond T.J., Berger I. (2006). Protein Complex Expression by Using Multigene Baculoviral Vectors. Nat. Methods.

[50] Bieniossek C., Richmond T.J., Berger I. (2008). MultiBac: Multigene Baculovirus-Based Eukaryotic Protein Complex Production. Curr. Protoc. Protein Sci..

[51] Bieniossek C., Imasaki T., Takagi Y., Berger I. (2012). MultiBac: Expanding the Research Toolbox for Multiprotein Complexes. Trends Biochem. Sci..

[52] Trowitzsch S., Palmberger D., Fitzgerald D., Takagi Y., Berger I. (2012). MultiBac Complexomics. Expert Rev. Proteom..

[53] Nie Y., Chaillet M., Becke C., Haffke M., Pelosse M., Fitzgerald D., Collinson I., Schaffitzel C., Berger I. (2016). ACEMBL Tool-Kits for High-Throughput Multigene Delivery and Expression in Prokaryotic and Eukaryotic Hosts. Adv. Exp. Med. Biol..

[54] Shang Y., Wang M., Xiao G., Wang X., Hou D., Pan K., Liu S., Li J., Wang J., Arif B.M. (2017). Construction and Rescue of a Functional Synthetic Baculovirus. ACS Synth. Biol..

[55] Shang Y., Hu H., Wang X., Wang H., Deng F., Wang M., Hu Z. (2021). Construction and Characterization of a Novel Bacmid AcBac-Syn Based on a Synthesized Baculovirus Genome. Virol. Sin..

[56] Thimiri Govinda Raj D.B., Garzoni F., Gavin A.C., Gibson T., Berger I. (2014). FEBS-EMBO Concurrent Session (Lecture): CS III-6-3 SynBac: Designer Minimal Baculovirus Genome for Drug Discovery. FEBS J..

[57] Adli M. (2018). The CRISPR Tool Kit for Genome Editing and Beyond. Nat. Commun..

[58] Hendriks D., Clevers H., Artegiani B. (2020). CRISPR-Cas Tools and Their Application in Genetic Engineering of Human Stem Cells and Organoids. Cell Stem Cell.

[59] Ishino Y., Shinagawa H., Makino K., Amemura M., Nakata A. (1987). Nucleotide Sequence of the Iap Gene, Responsible for Alkaline Phosphatase Isozyme Conversion in *Escherichia coli*, and Identification of the Gene Product. J. Bacteriol..

[60] Bolotin A., Quinquis B., Sorokin A., Ehrlich S.D. (2005). Clustered Regularly Interspaced Short Palindrome Repeats (CRISPRs) Have Spacers of Extrachromosomal Origin. Microbiology.

[61] Barrangou R., Fremaux C., Deveau H., Richards M., Boyaval P., Moineau S., Romero D.A., Horvath P. (2007). CRISPR Provides Acquired Resistance against Viruses in Prokaryotes. Science.

[62] Jinek M., Chylinski K., Fonfara I., Hauer M., Doudna J.A., Charpentier E. (2012). A Programmable Dual-RNA-Guided DNA Endonuclease in Adaptive Bacterial Immunity. Science.

[63] Wiedenheft B., Sternberg S.H., Doudna J.A. (2012). RNA-Guided Genetic Silencing Systems in Bacteria and Archaea. Nature.

[64] Hsu P.D., Lander E.S., Zhang F. (2014). Development and Applications of CRISPR-Cas9 for Genome Engineering. Cell.

[65] Pickar-Oliver A., Gersbach C.A. (2019). The next Generation of CRISPR-Cas Technologies and Applications. Nat. Rev. Mol. Cell Biol..

[66] Katti A., Diaz B.J., Caragine C.M., Sanjana N.E., Dow L.E. (2022). CRISPR in Cancer Biology and Therapy. Nat. Rev. Cancer.

[67] Hille F., Richter H., Wong S.P., Bratovič M., Ressel S., Charpentier E. (2018). The Biology of CRISPR-Cas: Backward and Forward. Cell.

[68] Koonin E.V., Makarova K.S. (2019). Origins and Evolution of CRISPR-Cas Systems. Philos. Trans. R. Soc. Lond. Ser. B Biol. Sci..

[69] Makarova K.S., Wolf Y.I., Iranzo J., Shmakov S.A., Alkhnbashi O.S., Brouns S.J.J., Charpentier E., Cheng D., Haft D.H., Horvath P. (2020). Evolutionary Classification of CRISPR-Cas Systems: A Burst of Class 2 and Derived Variants. Nat. Rev. Microbiol..

[70] Liu C., Zhang L., Liu H., Cheng K. (2017). Delivery Strategies of the CRISPR-Cas9 Gene-Editing System for Therapeutic Applications. J. Control. Release.

[71] Sander J.D., Joung J.K. (2014). CRISPR-Cas Systems for Editing, Regulating and Targeting Genomes. Nat. Biotechnol..

[72] Sternberg S.H., Redding S., Jinek M., Greene E.C., Doudna J.A. (2014). DNA Interrogation by the CRISPR RNA-Guided Endonuclease Cas9. Nature.

[73] Cong L., Ran F.A., Cox D., Lin S., Barretto R., Habib N., Hsu P.D., Wu X., Jiang W., Marraffini L.A. (2013). Multiplex Genome Engineering Using CRISPR/Cas Systems. Science.

[74] Jinek M., East A., Cheng A., Lin S., Ma E., Doudna J. (2013). RNA-Programmed Genome Editing in Human Cells. Elife.

[75] Mali P., Yang L., Esvelt K.M., Aach J., Guell M., DiCarlo J.E., Norville J.E., Church G.M. (2013). RNA-Guided Human Genome Engineering via Cas9. Science.

[76] Frock R.L., Hu J., Meyers R.M., Ho Y.-J., Kii E., Alt F.W. (2015). Genome-Wide Detection of DNA Double-Stranded Breaks Induced by Engineered Nucleases. Nat. Biotechnol..

[77] Su T., Liu F., Gu P., Jin H., Chang Y., Wang Q., Liang Q., Qi Q. (2016). A CRISPR-Cas9 Assisted Non-Homologous End-Joining Strategy for One-Step Engineering of Bacterial Genome. Sci. Rep..

[78] Brinkman E.K., Chen T., de Haas M., Holland H.A., Akhtar W., van Steensel B. (2018). Kinetics and Fidelity of the Repair of Cas9-Induced Double-Strand DNA Breaks. Mol. Cell.

[79] Schep R., Brinkman E.K., Leemans C., Vergara X., van der Weide R.H., Morris B., van Schaik T., Manzo S.G., Peric-Hupkes D., van den Berg J. (2021). Impact of Chromatin Context on Cas9-Induced DNA Double-Strand Break Repair Pathway Balance. Mol. Cell.

[80] Nambiar T.S., Baudrier L., Billon P., Ciccia A. (2022). CRISPR-Based Genome Editing through the Lens of DNA Repair. Mol. Cell.

[81] Qi L.S., Larson M.H., Gilbert L.A., Doudna J.A., Weissman J.S., Arkin A.P., Lim W.A. (2013). Repurposing CRISPR as an RNA-Guided Platform for Sequence-Specific Control of Gene Expression. Cell.

[82] Shariati S.A., Dominguez A., Xie S., Wernig M., Qi L.S., Skotheim J.M. (2019). Reversible Disruption of Specific Transcription Factor-DNA Interactions Using CRISPR/Cas9. Mol. Cell.

[83] Moradpour M., Abdulah S.N.A. (2020). CRISPR/DCas9 Platforms in Plants: Strategies and Applications beyond Genome Editing. Plant Biotechnol. J..

[84] Brezgin S., Kostyusheva A., Kostyushev D., Chulanov V. (2019). Dead Cas Systems: Types, Principles, and Applications. Int. J. Mol. Sci..

[85] Saifaldeen M., Al-Ansari D.E., Ramotar D., Aouida M. (2020). CRISPR FokI Dead Cas9 System: Principles and Applications in Genome Engineering. Cells.

[86] Karlson C.K.S., Mohd-Noor S.N., Nolte N., Tan B.C. (2021). CRISPR/DCas9-Based Systems: Mechanisms and Applications in Plant Sciences. Plants.

[87] Boettcher M., McManus M.T. (2015). Choosing the Right Tool for the Job: RNAi, TALEN, or CRISPR. Mol. Cell.

[88] Xu X., Qi L.S. (2019). A CRISPR-DCas Toolbox for Genetic Engineering and Synthetic Biology. J. Mol. Biol..

[89] Aulicino F., Pelosse M., Toelzer C., Capin J., Ilegems E., Meysami P., Rollarson R., Berggren P.O., Dillingham M.S., Schaffitzel C. (2022). Highly Efficient CRISPR-Mediated Large DNA Docking and Multiplexed Prime Editing Using a Single Baculovirus. Nucleic Acids Res..

[90] Cox D.B.T., Platt R.J., Zhang F. (2015). Therapeutic Genome Editing: Prospects and Challenges. Nat. Med..

[91] Barrangou R., Doudna J.A. (2016). Applications of CRISPR Technologies in Research and Beyond. Nat. Biotechnol..

[92] Zhou Q., Krebs J.F., Snipas S.J., Price A., Alnemri E.S., Tomaselli K.J., Salvesen G.S. (1998). Interaction of the Baculovirus Anti-Apoptotic Protein P35 with Caspases. Specificity, Kinetics, and Characterization of the Caspase/P35 Complex. Biochemistry.

[93] Lo W.D., Akhmametyeva E.M., Zhu L., Friesen P.D., Chang L.-S. (2003). Induction of Apoptosis by the P53-Related P73 and Partial Inhibition by the Baculovirus-Encoded P35 in Neuronal Cells. Mol. Brain Res..

[94] Rong R., Li T., Zhang Y., Gu Y., Xia N., Li S. (2019). Progress in Vaccine Development Based on Baculovirus Expression Vector System. Sheng Wu Gong Cheng Xue Bao.

[95] Dong Z., Qin Q., Hu Z., Chen P., Huang L., Zhang X., Tian T., Lu C., Pan M. (2019). Construction of a One-Vector Multiplex CRISPR/Cas9 Editing System to Inhibit Nucleopolyhedrovirus Replication in Silkworms. Virol. Sin..

[96] Bruder M.R., Walji S.-D., Aucoin M.G. (2021). Comparison of CRISPR–Cas9 Tools for Transcriptional Repression and Gene Disruption in the BEVS. Viruses.

[97] Pazmiño-Ibarra V., Mengual-Martí A., Targovnik A.M., Herrero S. (2019). Improvement of Baculovirus as Protein Expression Vector and as Biopesticide by CRISPR/Cas9 Editing. Biotechnol. Bioeng..

[98] Chen X., Sun X., Hu Z., Li M., O’Reilly D.R., Zuidema D., Vlak J.M. (2000). Genetic Engineering of *Helicoverpa armigera* Single-Nucleocapsid Nucleopolyhedrovirus as an Improved Pesticide. J. Invertebr. Pathol..

[99] Cory J.S., Clarke E.E., Brown M.L., Hails R.S., O’Reilly D.R. (2004). Microparasite Manipulation of an Insect: The Influence of the Egt Gene on the Interaction between a Baculovirus and Its Lepidopteran Host. Funct. Ecol..

[100] Georgievska L., Hoover K., van der Werf W., Muñoz D., Caballero P., Cory J.S., Vlak J.M. (2010). Dose Dependency of Time to Death in Single and Mixed Infections with a Wildtype and Egt Deletion Strain of *Helicoverpa armigera* Nucleopolyhedrovirus. J. Invertebr. Pathol..

[101] Simón O., Williams T., López-Ferber M., Caballero P. (2012). Deletion of Egt Is Responsible for the Fast-Killing Phenotype of Natural Deletion Genotypes in a *Spodoptera frugiperda* Multiple Nucleopolyhedrovirus Population. J. Invertebr. Pathol..

[102] Antoniou P., Miccio A., Brusson M. (2021). Base and Prime Editing Technologies for Blood Disorders. Front. Genome Ed..

[103] Anzalone A.V., Randolph P.B., Davis J.R., Sousa A.A., Koblan L.W., Levy J.M., Chen P.J., Wilson C., Newby G.A., Raguram A. (2019). Search-and-Replace Genome Editing without Double-Strand Breaks or Donor DNA. Nature.

[104] Schwinn M.K., Machleidt T., Zimmerman K., Eggers C.T., Dixon A.S., Hurst R., Hall M.P., Encell L.P., Binkowski B.F., Wood K.V. (2018). CRISPR-Mediated Tagging of Endogenous Proteins with a Luminescent Peptide. ACS Chem. Biol..

[105] García-Fernández A., Vivo-Llorca G., Sancho M., García-Jareño A.B., Ramírez-Jiménez L., Barber-Cano E., Murguía J.R., Orzáez M., Sancenón F., Martínez-Máñez R. (2022). Nanodevices for the Efficient Codelivery of CRISPR-Cas9 Editing Machinery and an Entrapped Cargo: A Proposal for Dual Anti-Inflammatory Therapy. Pharmaceutics.

[106] Aulicino F., Capin J., Berger I. (2020). Synthetic Virus-Derived Nanosystems (Svns) for Delivery and Precision Docking of Large Multifunctional Dna Circuitry in Mammalian Cells. Pharmaceutics.

[107] Mansouri M., Berger P. (2018). Baculovirus for Gene Delivery to Mammalian Cells: Past, Present and Future. Plasmid.

[108] Khan A.H., Bayat H., Rajabibazl M., Sabri S., Rahimpour A. (2017). Humanizing Glycosylation Pathways in Eukaryotic Expression Systems. World J. Microbiol. Biotechnol..

[109] Jacobs P.P., Callewaert N. (2009). N-Glycosylation Engineering of Biopharmaceutical Expression Systems. Curr. Mol. Med..

[110] Dalziel M., Crispin M., Scanlan C.N., Zitzmann N., Dwek R.A. (2014). Emerging Principles for the Therapeutic Exploitation of Glycosylation. Science.

[111] Wormald M.R., Dwek R.A. (1999). Glycoproteins: Glycan Presentation and Protein-Fold Stability. Structure.

[112] Bishop J.R., Schuksz M., Esko J.D. (2007). Heparan Sulphate Proteoglycans Fine-Tune Mammalian Physiology. Nature.

[113] Varki A. (2008). Sialic Acids in Human Health and Disease. Trends Mol. Med..

[114] Sperandio M., Gleissner C.A., Ley K. (2009). Glycosylation in Immune Cell Trafficking. Immunol. Rev..

[115] Marchal I., Jarvis D.L., Cacan R., Verbert A. (2001). Glycoproteins from Insect Cells: Sialylated or Not?. Biol. Chem..

[116] Geisler C., Jarvis D.L. (2012). Innovative Use of a Bacterial Enzyme Involved in Sialic Acid Degradation to Initiate Sialic Acid Biosynthesis in Glycoengineered Insect Cells. Metab. Eng..

[117] Kim A., Lee G., Hwang J.-H., Park J.-H., Lee M.J., Kim B., Kim S.-M. (2022). BacMam Expressing Highly Glycosylated Porcine Interferon Alpha Induces Robust Antiviral and Adjuvant Effects against Foot-and-Mouth Disease Virus in Pigs. J. Virol..

[118] Contreras-Gómez A., Sánchez-Mirón A., García-Camacho F., Molina-Grima E., Chisti Y. (2014). Protein Production Using the Baculovirus-Insect Cell Expression System. Biotechnol. Prog..

[119] Palomares L.A., Srivastava I.K., Ramírez O.T., Cox M.M.J. (2021). Glycobiotechnology of the Insect Cell-Baculovirus Expression System Technology. Adv. Biochem. Eng. Biotechnol..

[120] Legardinier S., Klett D., Poirier J.-C., Combarnous Y., Cahoreau C. (2005). Mammalian-like Nonsialyl Complex-Type N-Glycosylation of Equine Gonadotropins in Mimic Insect Cells. Glycobiology.

[121] Hollister J., Grabenhorst E., Nimtz M., Conradt H., Jarvis D.L. (2002). Engineering the Protein N-Glycosylation Pathway in Insect Cells for Production of Biantennary, Complex N-Glycans. Biochemistry.

[122] Hollister J.R., Jarvis D.L. (2001). Engineering Lepidopteran Insect Cells for Sialoglycoprotein Production by Genetic Transformation with Mammalian Beta 1,4-Galactosyltransferase and Alpha 2,6-Sialyltransferase Genes. Glycobiology.

[123] Aumiller J.J., Hollister J.R., Jarvis D.L. (2003). A Transgenic Insect Cell Line Engineered to Produce CMP-Sialic Acid and Sialylated Glycoproteins. Glycobiology.

[124] Aumiller J.J., Mabashi-Asazuma H., Hillar A., Shi X., Jarvis D.L. (2012). A New Glycoengineered Insect Cell Line with an Inducibly Mammalianized Protein N-Glycosylation Pathway. Glycobiology.

[125] Mabashi-Asazuma H., Shi X., Geisler C., Kuo C.-W., Khoo K.-H., Jarvis D.L. (2013). Impact of a Human CMP-Sialic Acid Transporter on Recombinant Glycoprotein Sialylation in Glycoengineered Insect Cells. Glycobiology.

[126] Mabashi-Asazuma H., Jarvis D.L. (2017). CRISPR-Cas9 Vectors for Genome Editing and Host Engineering in the Baculovirus–Insect Cell System. Proc. Natl. Acad. Sci. USA.

[127] Nweke E.E., Thimiri Govinda Raj D.B. (2021). Development of Insect Cell Line Using CRISPR Technology. Prog. Mol. Biol. Transl. Sci..

[128] Geisler C., Aumiller J.J., Jarvis D.L. (2008). A Fused Lobes Gene Encodes the Processing Beta-N-Acetylglucosaminidase in Sf9 Cells. J. Biol. Chem..

[129] Kim N.Y., Baek J.Y., Choi H.S., Chung I.S., Shin S., Lee J.I., Choi J.Y., Yang J.M. (2012). Short-Hairpin RNA-Mediated Gene Expression Interference in Trichoplusia Ni Cells. J. Microbiol. Biotechnol..

[130] Mabashi-Asazuma H., Kuo C.-W., Khoo K.-H., Jarvis D.L. (2015). Modifying an Insect Cell N-Glycan Processing Pathway Using CRISPR-Cas Technology. ACS Chem. Biol..

[131] Chavez-Pena C., Kamen A.A. (2018). RNA Interference Technology to Improve the Baculovirus-Insect Cell Expression System. Biotechnol. Adv..

